# The age-related changes of dietary phosphate responsiveness in plasma 1,25-dihydroxyvitamin D levels and renal Cyp27b1 and Cyp24a1 gene expression is associated with renal α-Klotho gene expression in mice

**DOI:** 10.3164/jcbn.17-20

**Published:** 2017-11-28

**Authors:** Ryouhei Yoshikawa, Hironori Yamamoto, Otoki Nakahashi, Tomohiro Kagawa, Mari Tajiri, Mari Nakao, Shiori Fukuda, Hidekazu Arai, Masashi Masuda, Masayuki Iwano, Eiji Takeda, Yutaka Taketani

**Affiliations:** 1 Department of Clinical Nutrition and Food Management, Institute of Biomedical Sciences, University of Tokushima Graduate School, 3-18-15 Kuramoto-cho, Tokushima 770-8503, Japan; 2 Department of Health and Nutrition, Faculty of Human Life, Jin-ai University, 3-1-1 Ohde-cho, Echizen-city, Fukui 915-8586, Japan; 3 Department of Nephrology, Faculty of Medical Sciences, University of Fukui, 23-3 Matsuoka Shimoaizuki, Eiheiji-cho, Yoshida-gun, Fukui 910-1193, Japan; 4 Division of Functional Food Chemistry, Institute for Health Science, Tokushima Bunri University, 180 Nishihamahoji, Yamashiro-cho, Tokushima 770-8514, Japan; 5 Laboratory of Clinical Nutrition and Management, Graduate School of Nutritional and Environmental Sciences, The University of Shizuoka, 52-1 Yada, Suruga-ku, Shizuoka 422-8526, Japan

**Keywords:** α-Klotho, age-related change, vitamin D metabolism, inorganic phosphate, mice

## Abstract

In this study, we investigated the relationship between age-related changes in renal α-Klotho gene expression, vitamin D metabolism and the responsiveness of dietary phosphate in 1, 2 and 13 month-old mice fed a high phosphate (phosphate 1.2%) diet or low phosphate (phosphate 0.02%) diet for 5 days. We found that 1,25-dihydroxyvitamin D levels in plasma were significantly lower in the high phosphate group than the low phosphate group for 1 and 2 month-old mice, but not 13 month-old mice. In addition, in the high phosphate group plasma 1,25-dihydroxyvitamin D levels were decreased in 2 month-old mice relative to 1 month-old mice, but 13 month-old mice had higher levels than 2 month-old mice. In fact, plasma 1,25-dihydroxyvitamin D levels showed a significant correlation with vitamin D metabolism gene *Cyp27b1* and *Cyp24a1* mRNA expression in the high phosphate group. Interestingly, renal α*-Klotho* mRNA and protein levels were significant change with age. Furthermore, α*-Klotho* mRNA expression showed a significant negative correlation with plasma 1,25-dihydroxyvitamin D levels in the high phosphate group. Our results suggest that age-related alterations in renal α-Klotho expression could affect the responsiveness of dietary phosphate to vitamin D metabolism.

## Introduction

Vitamin D can be obtained from the diet and synthesized from 7-dehydrocholesterol in the skin in response to sunlight.^(1,2)^ The first step in the metabolic activation of vitamin D is synthesis of 25-dihydroxyvitamin D [25(OH)D] by 25-hydroxylase, which occurs primarily in the liver. The second step, the formation of 1,25-dihydroxyvitamin D [1,25(OH)_2_D] from 25(OH)D, is catalyzed by the mitochondrial cytochrome P450 enzyme 25-dihydroxyvitamin D 1α-hydroxylase (encoded by the *Cyp27b1* gene) under physiological conditions, mainly in the kidney.^(2,3)^ 1,25(OH)_2_D has physiological activity, and is involved in regulating calcium (Ca) homeostasis via transcriptional control of genes in the relevant organ mediated by binding to the vitamin D receptor (VDR).^(4)^ In addition to conversion to 1,25(OH)_2_D by Cyp27b1 in the kidney, 25(OH)D can also be converted to 24,25-dihydroxyvitamin D [24,25(OH)_2_D] by the mitochondrial cytochrome P450 enzyme 25-dihydroxyvitamin D 24-hydroxylase encoded by the *Cyp24a1* gene.^(2)^ This enzyme can hydroxylate not only 25(OH)D but also 1,25(OH)_2_D and catabolizes these molecules into biologically inactive, water-soluble calcitroic acid.^(3,4)^

Phosphate (Pi) plays an important role in skeletal mineralization, mineral metabolism, and diverse cellular functions involving energy metabolism.^(5,6)^ Pi homeostasis is maintained by regulation of dietary absorption, bone formation, and renal excretion.^(7)^ The crucial regulated step in Pi homoeostasis is the transport of Pi in the brush-border membrane (BBM) of the renal proximal tubule. Renal Pi transport is mediated by several sodium-dependent Pi co-transporters (Npts), which have been classified into three categories: type I (Npt1), type II (Npt2a and Npt2c) and type III (PiT1 and PiT2).^(5,6)^ Pi homeostasis is regulated by several endocrine factors such as 1,25(OH)_2_D and parathyroid hormone (PTH), which are also important regulators of Ca homeostasis.^(1,8,9)^ In addition, recent studies have identified fibroblast growth factor (FGF) 23 as a new regulator of Pi metabolism.^(10)^

Aging is a progressive and unavoidable biological process involving malfunction of many tissue and organ. Aging is associated with modification of several organ functions and homeostatic adaptation, including vitamin D and Pi metabolism.^(11,12)^ The α-Klotho (Klotho) gene was identified as an “anti-aging” gene when its disruption in mice was found to induce a phenotype that mimicked age-related symptoms, including atrophy of skin, thymus and muscles, osteoporosis, ectopic calcification, diminished hearing, pulmonary emphysema and pituitary gland abnormalities.^(13)^ In contrast, mice that overexpress Klotho had increased life-spans.^(14)^ The Klotho gene encodes a single pass transmembrane protein and is expressed in multiple tissues, but Klotho levels in the kidney are particularly high.^(13,15)^ In addition to the membrane-bound form of Klotho, a secreted form of the Klotho protein is generated from the Klotho gene through alternative splicing. Secreted form of the Klotho is released directly into the extracellular domain and is present in body fluid. Another form of secreted Klotho in body fluid arises from the extracellular domain that is shed from the membrane form of Klotho expressed on cell surfaces by the protease a disintegrin and metalloproteinase 10/17 (ADAM 10/17).^(16,17)^

After the discovery of Klotho and FGF23, Klotho was found to form a complex with several FGF receptor (FGFR) isoforms (FGFR1c, 3c and 4). Binding of FGF23 to the FGFR/Klotho complex is necessary in order to activate the FGF23 signaling pathway, which is referred to as the FGF23/Klotho system.^(18)^ In addition, several lines of evidence indicate that the FGF23/Klotho system down-regulates 1,25(OH)_2_D and Pi levels by suppressing expression of Cyp27b1, Npt2a and Npt2c, while promoting Cyp24a1 expression in the kidney when plasma Pi concentration is high.^(19–21)^ Indeed, Klotho mutant mice (*kl/kl* mice) show hypervitaminosis D and hyperphosphatemia.^(13)^ Interestingly, various aging-like symptoms seen in *kl/kl* mice could be ameliorated by dietary restriction of vitamin D,^(22)^ genetic ablation of the *Cyp27b1* or *Vdr* gene,^(23,24)^ a Pi deficient diet^(25)^ or *Npt2a* and *Klotho* double-knockout.^(26)^ These reports suggest that vitamin D and Pi metabolism disorders are linked in these mice.

Klotho gene expression is affected by several factors and in various diseases, including dietary 1,25(OH)_2_D,^(22)^ Pi,^(25)^ oxidative stress,^(27)^ senescence,^(28)^ chronic kidney disease (CKD)^(29,30)^ and diabetes.^(31)^ However, whether changes in Klotho gene expression with age affect vitamin D metabolism is unclear. In this study, we investigated the relationship between various age-related changes, including renal Klotho gene expression and vitamin D metabolism, and the responsiveness of dietary Pi using 1, 2 and 13 month-old mice fed a high or low Pi diet.

## Materials and Methods

### Animals and diets

Male C57BL/6J mice were purchased from Japan SLC (Hamamatsu, Japan). All animals were kept on a 12 h:12 h light-dark cycle (lights on at 8 AM, lights off at 8 PM) with normal diet and water freely available. Mice were examined at 1, 2 and 13 months of age. In addition, 1 month-old mice were weaned at 3 weeks of age. In each age group, mice were divided into two groups as follows: High Pi diet (HP: 1.2% Pi, 0.6% Ca) group and Low Pi diet (LP: 0.02% Pi, 0.6% Ca) group. Following 2 days intake of the control Pi diet (CP: 0.6% Pi, 0.6% Ca), the mice were fed the HP diet or LP diet for 5 days and then sacrificed. All of the diets used in each experiment had a modified AIN-93G composition (Table 1).^(32)^ The breeding and handling of all animals in these experiments was approved by the University of Tokushima Animal Experimentation Committee.

### Blood and urine parameters

The concentrations of inorganic Pi, Ca and creatinine (Cre) were determined using the Phospha C-test Wako, the Calcium E-test Wako, and LabAssay^TM^ Creatinine test kits, respectively (Wako, Osaka, Japan). Concentrations of intact FGF23 and intact PTH (1-84) in the plasma were determined using the FGF23 ELISA Kit (Kinos, Tokyo, Japan) and Mouse PTH 1-84 ELISA Kit (Immutopics, San Clemente, CA), respectively. Plasma 1,25(OH)_2_D concentrations were determined by radioimmunoassay (SRL, Tokyo, Japan).

### Real-time RT-PCR analysis

Total RNA was isolated from kidneys using RNAiso plus reagent (Takara Bio, Shiga, Japan) and from femurs using ISOGEN reagent (Nippon Gene, Tokyo, Japan). Extracted total RNA was treated with DNase І. First-strand cDNA was synthesized from 2.5 µg of total RNA, primed with oligo (dT) using the M-MLV-reverse transcriptase (Invitrogen, Carlsbad, CA). The first-strand cDNA was PCR amplified using SYBER Select Master Mix (Applied Biosystems, Foster City, CA) in a 20 µl reaction volume, with 4 pmol of each primer (Table 2). Real-time PCR was performed with an Applied Biosystems Step one plus thermocycler (Applied Biosystems, Foster City, CA). The PCR products were quantified by fit point analysis and results were normalized to those for *β-actin*.

### Western blot analysis

Kidney tissue was homogenized in lysis buffer (1% Triton X-100, 50 mM Tris-HCl, 150 mM NaCl, 5 mM EDTA), incubated on ice for 10 min, and centrifuged at 1,000 rpm for 10 min. Equal protein loading was verified by Bradford assay using the Bio-Rad Protein Assay (Bio-Rad, CA). Supernatants were incubated with 2× sodium dodecyl sulfate (SDS) sample buffer, separated on 10% SDS-polyacrylamide gels, and electroblotted onto polyvinylidene difluoride membranes (Immobilon-P, Millipore, Billerica, MA). Membranes were blocked at room temperature with 5% non-fat dried milk in phosphate buffered saline (PBS) containing 0.05% Tween-20 (PBS-t). The following primary antibodies were used: anti-human klotho monoclonal antibody KM2076 (1:250 dilution) (Trans Genic, Kumamoto, Japan) and mouse anti-actin monoclonal antibody (1:2,000 dilution) (Sigma-Aldrich). Secondary antibodies were: goat anti-rat IgG (H + L) antibody conjugated to HRP (1:5,000 dilution) (Cell Signaling Technologies) and goat anti-mouse IgG (H + L) HRP conjugate (1:5,000 dilution) (Invitrogen). Membranes were incubated with the primary antibodies overnight at 4°C, washed three times with PBS-t, and incubated with the secondary antibodies for 1 h at room temperature. Signals were detected using the Enhanced Chemiluminescense (ECL) Plus system (GE Healthcare Japan, Tokyo, Japan) and BioMax MR Film (Kodak Japan, Ltd., Tokyo, Japan).

### Statistical analysis

Data are expressed as means ± SEM. Unpaired *t* test and one-way ANOVA with a post-hoc test of either the Tukey-Kramer test were used for statistical comparisons between experimental groups. Pearson’s correlation coefficient analysis was used to assess the relationship between each parameter. *P*<0.05 was considered significant.

## Results

### Effects of dietary Pi on plasma and urine Pi and Ca levels in each age group mice

We measured plasma and urine Pi and Ca levels in 1, 2 and 13 month-old C57BL/6J mice that were fed either a HP or LP diet for 5 days (Table 3). Plasma Pi levels were significantly higher in the HP group than in the LP group at all ages. In particular, in the HP group the levels were significantly higher in the 1 month-old mice than the 2 and 13 month-old mice. In contrast, plasma Ca levels were significantly lower in the HP group than in 1 and 13 month-old LP group mice. Urinary Pi excretions were significantly higher in the HP group than the LP group at all ages, and were significantly higher for 1 month-old HP mice than 2 and 13 month-old HP mice. In contrast, urinary Ca excretions were significantly lower in the HP group than in 1 and 2 month-old LP mice, and in the HP group the 1 month-old animals had significantly higher levels relative to 2 and 13 month-old mice.

### Effects of dietary Pi on plasma 1,25(OH)_2_D, PTH and FGF23 levels in each age group mice

We next measured plasma 1,25(OH)_2_D, PTH and FGF23 levels in mice (Table 4). Plasma 1,25(OH)_2_D levels were significantly lower in the HP group than in the LP group for 1 and 2 month-old mice, but there was no significant difference between the HP and LP groups for 13 month-old mice. In the HP group, plasma 1,25(OH)_2_D levels were significantly decreased between 1 to 2 month-old mice, and were significantly increased for 13 month-old mice relative to 2 month-old mice. Plasma PTH and FGF23 levels were higher in the HP group than in the LP group at all ages, and only plasma FGF23 levels were significantly higher in 13 month-old mice compared to 1 month-old mice in the LP group.

### Effects of dietary Pi on vitamin D metabolism-related gene expression in each age group mice

**Fig. 1:**
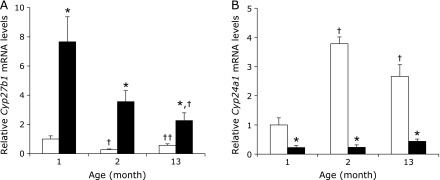
Effects of dietary Pi on renal *Cyp27b1* and *Cyp24a1* gene expression in each age group mice. 1, 2 and 13 month-old C57BL/6J mice were fed either a HP (□) or LP (■) diet for 5 days. Total RNA from the kidneys was extracted and gene expression was measured by quantitative real-time RT-PCR analysis. (A) Renal *Cyp27b1* mRNA expression. (B) Renal *Cyp24a1* mRNA expression levels. Data are expressed as means ± SEM (*n* = 4–5). *p<0.05 vs same age in the HP group, ^†^*p*<0.05 vs 1 month-old group, ^††^*p*<0.05 vs 2 month-old group.

Given the age-related changes seen in plasma 1,25(OH)_2_D levels, we next analyzed the mRNA expression levels of the vitamin D metabolizing enzymes *Cyp27b1* and *Cyp24a1* in mice kidney tissues (Fig. 1). For all age groups, renal *Cyp27b1* mRNA expression levels were significantly lower in the HP group compared to the LP group (Fig. 1A). In addition, in the LP group the renal *Cyp27b1* mRNA levels were significantly higher for 1 month-old mice compared to 13 month-old mice. Interestingly, in the HP group, *Cyp27b1* mRNA expression levels in 2 month-old mice were decreased relative to 1 month-old mice, yet the 13 month-old mice had levels that were increased with respect to 2 month-old mice. On the other hand, renal *Cyp24a1* mRNA expression levels were significantly higher in the HP group than in the LP group at all ages (Fig. 1B). Furthermore, in the HP group, *Cyp24a1* mRNA expression levels were increased in 2 month-old mice compared to 1 month-old mice, whereas 2 month-old mice had higher levels than 13 month-old mice. Moreover, *Cyp27b1* mRNA expression levels was positively correlated with plasma 1,25(OH)_2_D levels (r = 0.660, *p*<0.05) and *Cyp24a1* mRNA expression was negatively correlated with plasma 1,25(OH)_2_D levels (r = −0.722, *p*<0.05) in the HP group (data not shown).

### Effects of dietary Pi on FGF23/Klotho system-related gene expression in each age group mice

**Fig. 2:**
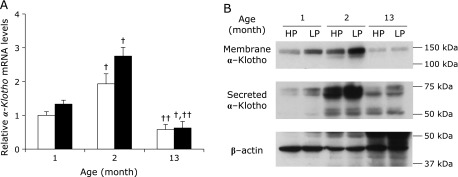
Effects of dietary Pi on renal α-Klotho gene expression in each age group mice. 1, 2 and 13 month-old C57BL/6J mice were fed either a HP (□) or LP (■) diet for 5 days. Total RNA from the kidneys was extracted and gene expression was measured by quantitative real-time RT-PCR analysis. (A) Renal *α-Klotho* mRNA expression. Data are expressed as means ± SEM (*n* = 4–5). ^†^*p*<0.05 vs 1 month-old group, ^††^*p*<0.05 vs 2 month-old group. Whole protein samples from mouse kidney tissues (20 µg) were analyzed by western blotting. (B) α-Klotho protein expression.

**Fig. 3:**
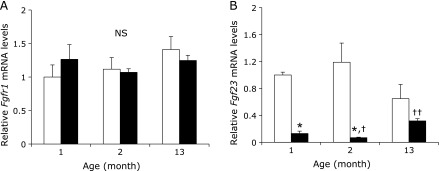
Effects of dietary Pi on renal *Fgfr1* and bone *Fgf23* gene expression in each age group mice. 1, 2 and 13 month-old C57BL/6J mice were fed either a HP (□) or LP (■) diet for 5 days. Mice kidney and bone total RNA was extracted and gene expression was measured by quantitative real-time RT-PCR analysis. (A) Renal *Fgfr1* mRNA expression. (B) Bone *Fgf23* mRNA expression levels. Data are expressed as means ± SEM (*n* = 4–9). *p<0.05 vs corresponding age in the HP group, ^†^*p*<0.05 vs 1 month-old group, ^††^*p*<0.05 vs 2 month-old group.

Since the FGF23/Klotho system is involved in regulating plasma 1,25(OH)_2_D levels and vitamin D metabolic enzymes, and this system is subject to age-related changes, we measured renal *Klotho* mRNA and protein expression levels (Fig. 2). Renal *Klotho* mRNA expression levels tended to be lower in the HP group than in the LP group for 1 and 2 month-old mice (Fig. 2A). Interestingly, these levels were significantly increased in 2 month-old mice compared to 1 month-old mice, and 13 month-old mice had significantly decreased levels compared to 2 month-old mice in both the HP and LP group. Similar to renal *Klotho* mRNA levels, membrane-associated and secreted Klotho protein expression levels in renal tissues from both the HP and LP groups were markedly increased in 2 month-old mice compared to 1 month-old mice, and 13 month-old mice had lower levels than did 2 month-old mice (Fig. 2B). Meanwhile, mRNA expression levels of renal *Fgfr1*, which forms a complex with Klotho, were not significantly different among the groups (Fig. 3A). In contrast, in bone *Fgf23* mRNA expression levels were significantly higher in the HP group than in the LP group at all ages and in the LP group levels decreased in the 2 month-old mice relative to 1 month-old mice, whereas the 13 month-old mice had higher levels than did 2 month-old mice (Fig. 3B).

### Relationship between renal *Klotho* mRNA expression, plasma 1,25(OH)_2_D levels and vitamin D metabolism-related gene expression

**Fig. 4:**
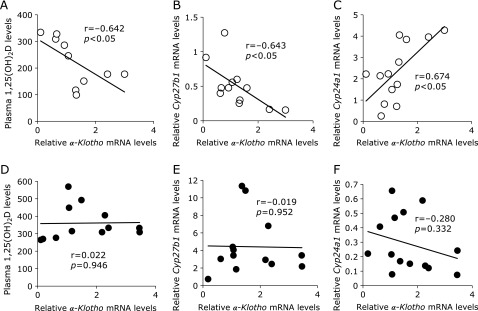
Correlation between renal *α-Klotho* mRNA expression and levels of plasma 1,25(OH)_2_D and FGF23/α-Klotho system target gene expression. 1, 2 and 13 month-old C57BL/6J mice were fed either a HP (◯) or LP (●) diet for 5 days. Correlation of renal *α-Klotho* mRNA levels with (A, D) plasma 1,25(OH)_2_D levels and renal (B,E) *Cyp27b1*, (C, F) *Cyp24a1* mRNA levels in each diet group (*n* = 10–14).

We next analyzed the relationship between *Klotho* mRNA expression and levels of plasma 1,25(OH)_2_D and vitamin D metabolic enzyme mRNA expression for all age and diet groups. In the HP group, plasma 1,25(OH)_2_D levels showed a negative correlation with *Klotho* mRNA expression (Fig. 4A). In addition, *Cyp27b1* mRNA expression was negatively correlated with *Klotho* mRNA expression (Fig. 4B) and *Cyp24a1* mRNA expression was positively correlated with *Klotho* mRNA expression in HP mice (Fig. 4C). In contrast, there was no correlation in the LP diet group (Fig. 4D–F).

## Discussion

In this study we investigated the relationship between age-related changes in renal Klotho gene expression, vitamin D metabolism and the responsiveness of dietary Pi in 1, 2 and 13 month-old mice fed a HP or LP diet for 5 days. Although defining “aging” in mice is difficult, for purposes of this study, we prepared the stage between 1 and 2 months as “growth”, and aging from 2 to 13 months was “maturation”. During this 12 month time period, we observed significant age-related changes in plasma 1,25(OH)_2_D levels and both *Klotho* mRNA and protein levels (Table 4, Fig. 2). FGF23 and PTH play critical roles in vitamin D and Pi homeostasis in the kidney, and respond to elevations in plasma Pi levels.^(8,10,33)^ Although in this study plasma FGF23 and PTH levels were significantly higher in the HP group than in the LP group, the levels of these two proteins were not significantly affected by age (Table 4). On the other hand, plasma 1,25(OH)_2_D levels did significantly change with age. Several reports showed that FGF23 reduces plasma 1,25(OH)_2_D concentration.^(34,35)^ Moreover, the FGF23/Klotho system is known to suppress *Cyp27b1* gene transcription, and stimulates transcription of the *Cyp24a1* gene through activating mitogen-activated protein kinase (MAPK) (ERK1/2) in cultured renal proximal tubular cells.^(36)^ Therefore, *kl/kl* mice and FGF23-deficient mice have hypervitaminosis D. Our data showed that with aging renal Klotho gene expression was significantly changed, but *Fgf23* and *Fgfr1* gene expression were essentially unchanged (Fig. 2 and 3). These results suggest that the cause of age-related changes in vitamin D metabolism could be attributed to changes in renal Klotho expression. In fact, renal *Klotho* mRNA expression had a significant negative correlation with *Cyp27b1* mRNA expression levels and plasma 1,25(OH)_2_D levels, and a positive correlation with *Cyp24a1* mRNA expression in the HP group (Fig. 4A–C). In addition, plasma PTH levels didn’t have a significant correlation with plasma 1,25(OH)_2_D levels in the HP group (data not shown), this result suggest that the FGF23/Klotho system might be more important than PTH on age-related changes in vitamin D metabolism.

Similar to results reported in previous studies,^(28,37)^ we observed a significant change in renal Klotho mRNA and protein levels with age (Fig. 2). The major inflammatory cytokine TNF-α was shown to reduce renal Klotho gene expression through a NFκB RelA-dependent mechanism.^(31,38)^ In this study, renal *Tnf-*α mRNA expression levels were significantly higher in 13 month-old mice relative to 2 month-old mice in both the HP and LP groups (data not shown). Increases in renal *Tnf-*α with maturation may be associated with decreased renal function. Our finding that *Tnf-*α mRNA levels were higher in older mice suggests that inflammation and reduced renal function can downregulate Klotho expression with maturation. On the other hand, our results showed that 2 month-old mice had higher Klotho gene expression levels than 1 month-old mice. In fact, renal Klotho expression is reported to be expressed at very low levels in newborn animals,^(15)^ suggesting that renal Klotho expression increases with growth. However, we observed reactivity of dietary Pi to plasma 1,25(OH)_2_D levels on the 1 month-old mice in spite of low levels of renal Klotho expression (Table 4). This result suggests that the renal Klotho expression levels in 1 month-old mice is sufficient in order to activate the FGF23 signaling pathway.

**Fig. 5:**
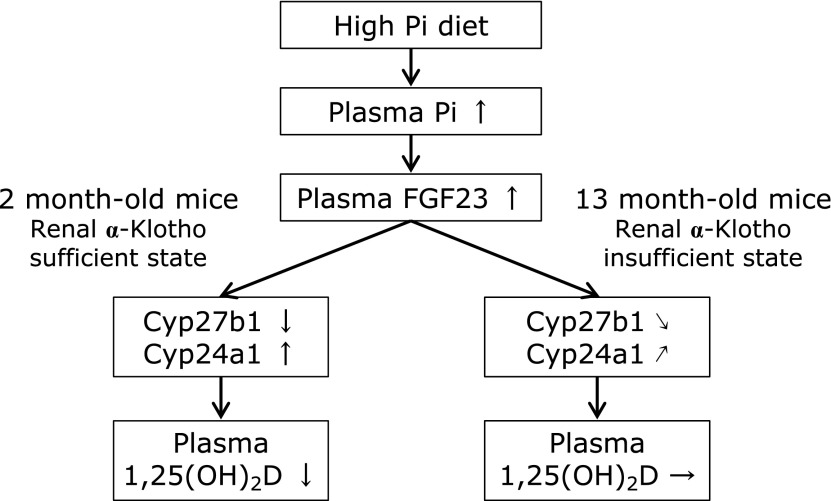
Age-related changes in renal α-Klotho gene expression could affect renal Cyp27b1 and Cyp24a1 gene expression and plasma 1,25(OH)_2_D levels upon intake of the HP diet. When the FGF23/α-Klotho system functions normally, HP diet intake increases plasma FGF23 levels, and decreases plasma 1,25(OH)_2_D levels. In this study, it was suggested that 13 month-old mice is FGF23/α-Klotho system malfunction state due to decrease of renal α-Klotho gene expression, and the 1,25(OH)_2_D synthesis inhibiting action by HP diet intake may be attenuated.

Recent studies have suggested that excess intake of Pi is a risk factor for the progression of CKD and cardiovascular disease (CVD).^(39–41)^ In addition, intake rates of Pi are increasing worldwide due to an increased consumption of processed foods.^(42,43)^ Notably, our data suggest that the FGF23/Klotho system in 13 month-old mice is malfunction state due to the low level of renal Klotho expression (Fig. 5). Abnormalities in FGF23/Klotho system function can induce abnormal vitamin D and Pi metabolism, as well as dietary Pi toxicity in response to excess dietary Pi. Higher levels of serum Pi within the normal range are associated with increased risk of ectopic calcification among individuals having a glomerular filtration rate of 60 ml/min/1.73 m^2^, but this is not associated with CKD.^(44)^ Therefore, many studies has focused on the relationship between serum Pi levels and the risk of death and other adverse clinical outcomes among individuals with normal renal function or with only moderate CKD.^(45–48)^ Normally, plasma 1,25(OH)_2_D levels are decreased in mice fed a HP diet.^(49)^ In the present study, plasma 1,25(OH)_2_D levels were significantly lower in the HP group relative to the LP group for 1 and 2 month-old mice, but there was no significant difference between the two groups for 13 month-old (Table 4). In addition, plasma 1,25(OH)_2_D levels and *Cyp27b1* mRNA expression were negatively correlated with *Klotho* mRNA expression and *Cyp24a1* mRNA expression was positively correlated with *Klotho* mRNA expression in the HP diet group, but there was no significant correlation between these factors in the LP diet group (Fig. 4). Increasing plasma 1,25(OH)_2_D levels induced by decreased levels of renal Klotho may lead to further hyperphosphatemia because 1,25(OH)_2_D increases plasma Pi concentrations. Further study will be needed to elucidate the association among Klotho gene expression, FGF23/Klotho system abnormality, vitamin D and Pi metabolism disorder and dietary Pi toxicity. Moreover, one of the limitations of this study is that we have not used the CP diet for comparison, so we cannot refer to the physiological changes in vitamin D metabolism and renal Klotho expression due to aging.

In summary, we observed significant changes in renal Klotho mRNA and protein expression with age. Furthermore, age-related changes in renal Klotho expression could affect gene expression of the vitamin D metabolizing enzymes *Cyp27b1* and *Cyp24a1*, and in turn plasma 1,25(OH)_2_D levels. In addition, an abnormal response to a high Pi diet because of reduced or insufficient renal Klotho expression may induce vitamin D disorder and dietary Pi toxicity. Finally, our results suggest that renal Klotho plays an important role for vitamin D and Pi homeostatic adaptations in growth and maturation. Improved knowledge of the relationship between aging and changes in renal Klotho expression in the context of abnormal FGF23/Klotho system function may reveal new possibilities for managing dietary Pi during various life stages.

## Figures and Tables

**Table 1:** Experimental diet compositions

Ingredient (g)	1.2% Pi (HP)	0.02% Pi (LP)
Egg white	20.0	20.0
l-Cysteine	0.3	0.3
Cornstarch	39.7	39.7
α-Cornstarch	13.2	13.2
Sugar	5.16	10.44
Soybean oil	7.0	7.0
Cellulose	5.0	5.0
Vitamin mix	1.0	1.0
Choline bitartrate	0.25	0.25
*tert*-Butylhydroquinone	0.0014	0.0014
CaCO_3_	1.4894	1.4894
KH_2_PO_4_	5.2731	0
Mineral mix changed	1.5645	1.5645

The mineral mix did not contain CaCO_3_ or KH_2_PO_4_.

**Table 2:** The primer sequences for PCR amplification

Gene name	Forward sequence (5' to 3')	Reverse sequence (5' to 3')	Gene accession NM
*Cyp27b1*	ATGGTGAAGAATGGCAGAGG	TAGTCGTCGCACAAGGTCAC	NM_010009
*Cyp24a1*	TCAAGCCAGCGTTCGGGTCTAA	TGCCATTCACAACTCGGACCCT	NM_009996
*α-Klotho*	CAAAAGCTGATAGAGGACAATGGC	GGCAGAGAAATCAACACAGTAAGG	NM_013823
*Fgfr1*	CCAGTCATCCATGAACTCTGGGGTTCTCC	GGTCACACGGTTGGGTTTGTCCTTATCCAG	NM_010206
*Fgf23*	ATGCTAGGGACCTGCCTTAGA	AGCCAAGCAATGGGGAAGTG	NM_022657
*β-actin*	CTGACCCTGAAGTACCCCATTGAACA	CTGGGGTGTTGAAGGTCTCAAACATG	NM_007393

**Table 3:** Effects of dietary Pi on plasma and urine Pi and Ca levels in each age group mice

	1 month			2 month			13 month	
	HP	LP		HP	LP		HP	LP
Plasma								
Pi (mg/dl)	9.2 ± 0.4	4.0 ± 0.2*		7.4 ± 0.3^†^	5.3 ± 0.6*		7.6 ± 0.4^†^	3.8 ± 0.5*
Ca (mg/dl)	8.2 ± 0.4	10.5 ± 0.6*		9.0 ± 0.2	9.8 ± 0.4		7.6 ± 0.4^††^	9.9 ± 0.6*
Urine								
Pi/Cre (mg/mgCre)	13.9 ± 1.6	0.08 ± 0.03*		7.8 ± 1.1^†^	0.1 ± 0.02*		7.5 ± 1.4^†^	0.03 ± 0.01*
Ca/Cre (mg/mgCre)	0.7 ± 0.1	4 ± 0.4*		0.3 ± 0.1^†^	1.3 ± 0.2*^,^^†^		0.2 ± 0.1^†^	0.7 ± 0.1^†^

1, 2 and 13 month-old C57BL/6J mice were fed either a HP or LP diet for 5 days. Data are expressed as means ± SEM (*n* = 4–10). *p<0.05 vs same age group in the HP group, ^†^*p*<0.05 vs 1 month-old group, ^††^*p*<0.05 vs 2 month-old group.

**Table 4:** Effects of dietary Pi on plasma 1,25(OH)_2_D, PTH and FGF23 levels in each age group mice

	1 month			2 month			13 month	
	HP	LP		HP	LP		HP	LP
1,25(OH)_2_D (pg/ml)	187 ± 22	507 ± 18*		138 ± 9^†^	270 ± 25*^,^^†^		314 ± 9^†^^,^^†^^†^	315 ± 31^†^
PTH (pg/ml)	17.8 ± 2.7	8.1 ± 1.2*		22.0 ± 2.6	7.6 ± 1.2*		21.4 ± 0.8	5.6 ± 1.4*
FGF23 (pg/ml)	310 ± 28	9 ± 2*		280 ± 19	27 ± 8*		247 ± 23	44 ± 10*^,^^†^

1, 2 and 13 month-old C57BL/6J mice were fed either a HP or LP diet for 5 days. Data are expressed as means ± SEM (*n* = 3–10). *p<0.05 vs same age group in the HP group, ^†^*p*<0.05 vs 1 month-old group, ^††^*p*<0.05 vs 2 month-old group.

## References

[1] Dusso AS, Brown AJ, Slatopolsky E (2005). Vitamin D. Am J Physiol Renal Physiol.

[2] Miller WL (2017). Genetic disorders of Vitamin D biosynthesis and degradation. J Steroid Biochem Mol Biol.

[3] Christakos S, Dhawan P, Verstuyf A, Verlinden L, Carmeliet G (2016). Vitamin D: Metabolism, molecular mechanism of action, and pleiotropic effects. Physiol Rev.

[4] Holick MF (2007). Vitamin D deficiency. N Engl J Med.

[5] Takeda E, Yamamoto H, Nashiki K, Sato T, Arai H, Taketani Y (2004). Inorganic phosphate homeostasis and the role of dietary phosphorus. J Cell Mol Med.

[6] Murer H, Hernando N, Forster I, Biber J (2000). Proximal tubular phosphate reabsorption: molecular mechanisms. Physiol Rev.

[7] Schiavi SC, Kumar R (2004). The phosphatonin pathway: new insights in phosphate homeostasis. Kidney Int.

[8] Berndt T, Kumar R (2007). Phosphatonins and the regulation of phosphate homeostasis. Annu Rev Physiol.

[9] Moor MB, Bonny O (2016). Ways of calcium reabsorption in the kidney. Am J Physiol Renal Physiol.

[10] Shimada T, Mizutani S, Muto T (2001). Cloning and characterization of FGF23 as a causative factor of tumor-induced osteomalacia. Proc Natl Acad Sci U S A.

[11] Fujisawa Y, Kida K, Matsuda H (1984). Role of change in vitamin D metabolism with age in calcium and phosphorus metabolism in normal human subjects. J Clin Endocrinol Metab.

[12] Chau TS, Lai WP, Cheung PY, Favus MJ, Wong MS (2005). Age-related alteration of vitamin D metabolism in response to low-phosphate diet in rats. Br J Nutr.

[13] Kuro-o M, Matsumura Y, Aizawa H (1997). Mutation of the mouse klotho gene leads to a syndrome resembling ageing. Nature.

[14] Kurosu H, Yamamoto M, Clark JD (2005). Suppression of aging in mice by the hormone Klotho. Science.

[15] Ohyama Y, Kurabayashi M, Masuda H (1998). Molecular cloning of rat klotho cDNA: markedly decreased expression of klotho by acute inflammatory stress. Biochem Biophys Res Commun.

[16] Hu MC, Kuro-o M, Moe OW (2013). Renal and extrarenal actions of Klotho. Semin Nephrol.

[17] Bian A, Neyra JA, Zhan M, Hu MC (2015). *Klotho*, stem cells, and aging. Clin Interv Aging.

[18] Kurosu H, Ogawa Y, Miyoshi M (2006). Regulation of fibroblast growth factor-23 signaling by klotho. J Biol Chem.

[19] Shimada T, Kakitani M, Yamazaki Y (2004). Targeted ablation of Fgf23 demonstrates an essential physiological role of FGF23 in phosphate and vitamin D metabolism. J Clin Invest.

[20] Segawa H, Yamanaka S, Ohno Y (2007). Correlation between hyperphosphatemia and type II Na-Pi cotransporter activity in klotho mice. Am J Physiol Renal Physiol.

[21] Shimada T, Hasegawa H, Yamazaki Y (2004). FGF-23 is a potent regulator of vitamin D metabolism and phosphate homeostasis. J Bone Miner Res.

[22] Tsujikawa H, Kurotaki Y, Fujimori T, Fukuda K, Nabeshima Y (2003). Klotho, a gene related to a syndrome resembling human premature aging, functions in a negative regulatory circuit of vitamin D endocrine system. Mol Endocrinol.

[23] Nabeshima Y (2009). Discovery of alpha-Klotho unveiled new insights into calcium and phosphate homeostasis. Proc Jpn Acad Ser B Phys Biol Sci.

[24] Ohnishi M, Nakatani T, Lanske B, Razzaque MS (2009). Reversal of mineral ion homeostasis and soft-tissue calcification of klotho knockout mice by deletion of vitamin D 1alpha-hydroxylase. Kidney Int.

[25] Morishita K, Shirai A, Kubota M (2001). The progression of aging in klotho mutant mice can be modified by dietary phosphorus and zinc. J Nutr.

[26] Ohnishi M, Razzaque MS (2010). Dietary and genetic evidence for phosphate toxicity accelerating mammalian aging. FASEB J.

[27] Mitobe M, Yoshida T, Sugiura H, Shirota S, Tsuchiya K, Nihei H (2005). Oxidative stress decreases klotho expression in a mouse kidney cell line. Nephron Exp Nephrol.

[28] Lim JH, Kim EN, Kim MY (2012). Age-associated molecular changes in the kidney in aged mice. Oxid Med Cell Longev.

[29] Hu MC, Shi M, Zhang J (2011). Klotho deficiency causes vascular calcification in chronic kidney disease. J Am Soc Nephrol.

[30] Sakan H, Nakatani K, Asai O (2014). Reduced renal α-Klotho expression in CKD patients and its effect on renal phosphate handling and vitamin D metabolism. PLoS One.

[31] Zhao Y, Banerjee S, Dey N (2011). Klotho depletion contributes to increased inflammation in kidney of the db/db mouse model of diabetes via RelA (serine)536 phosphorylation. Diabetes.

[32] Reeves PG (1997). Components of the AIN-93 diets as improvements in the AIN-76A diet. J Nutr.

[33] Hernández A, Concepción MT, Rodríguez M, Salido E, Torres A (1996). High phosphorus diet increases preproPTH mRNA independent of calcium and calcitriol in normal rats. Kidney Int.

[34] Quarles LD (2008). Endocrine functions of bone in mineral metabolism regulation. J Clin Invest.

[35] Cheng CY, Kuro-o M, Razzaque MS (2011). Molecular regulation of phosphate metabolism by fibroblast growth factor-23-klotho system. Adv Chronic Kidney Dis.

[36] Perwad F, Zhang MY, Tenenhouse HS, Portale AA (2007). Fibroblast growth factor 23 impairs phosphorus and vitamin D metabolism *in vivo* and suppresses 25-hydroxyvitamin D-1alpha-hydroxylase expression *in vitro*. Am J Physiol Renal Physiol.

[37] Yamazaki Y, Imura A, Urakawa I (2010). Establishment of sandwich ELISA for soluble alpha-Klotho measurement: age-dependent change of soluble alpha-Klotho levels in healthy subjects. Biochem Biophys Res Commun.

[38] Moreno JA, Izquierdo MC, Sanchez-Niño MD (2011). The inflammatory cytokines TWEAK and TNFα reduce renal klotho expression through NFκB. J Am Soc Nephrol.

[39] Jain N, Elsayed EF (2013). Dietary phosphate: what do we know about its toxicity. J Nephrol.

[40] Razzaque MS (2011). Phosphate toxicity: new insights into an old problem. Clin Sci (Lond).

[41] Calvo MS, Uribarri J (2013). Public health impact of dietary phosphorus excess on bone and cardiovascular health in the general population. Am J Clin Nutr.

[42] Uribarri J, Calvo MS (2014). Dietary phosphorus intake and health1-3. Am J Clin Nutr.

[43] Takeda E, Yamamoto H, Yamanaka-Okumura H, Taketani Y (2012). Dietary phosphorus in bone health and quality of life. Nutr Rev.

[44] Adeney KL, Siscovick DS, Ix JH (2009). Association of serum phosphate with vascular and valvular calcification in moderate CKD. J Am Soc Nephrol.

[45] Kestenbaum B, Sampson JN, Rudser KD (2005). Serum phosphate levels and mortality risk among people with chronic kidney disease. J Am Soc Nephrol.

[46] Narang R, Ridout D, Nonis C, Kooner JS (1997). Serum calcium, phosphorus and albumin levels in relation to the angiographic severity of coronary artery disease. Int J Cardiol.

[47] Tonelli M, Sacks F, Pfeffer M, Gao Z, Curhan G, Cholesterol And Recurrent Events Trial Investigators. (2005). Relation between serum phosphate level and cardiovascular event rate in people with coronary disease. Circulation.

[48] Dhingra R, Sullivan LM, Fox CS (2007). Relations of serum phosphorus and calcium levels to the incidence of cardiovascular disease in the community. Arch Intern Med.

[49] Azam N, Zhang MY, Wang X, Tenenhouse HS, Portale AA (2003). Disordered regulation of renal 25-hydroxyvitamin D-1alpha-hydroxylase gene expression by phosphorus in X-linked hypophosphatemic (hyp) mice. Endocrinology.

